# DECA: scalable XHMM exome copy-number variant calling with ADAM and Apache Spark

**DOI:** 10.1186/s12859-019-3108-7

**Published:** 2019-10-11

**Authors:** Michael D. Linderman, Davin Chia, Forrest Wallace, Frank A. Nothaft

**Affiliations:** 1 Department of Computer Science, Middlebury College, 75 Shannon St, Middlebury, VT 05753 USA; 2 AMPLab, University of California, Berkeley, Berkeley, CA USA; 3 Databricks, Inc., San Francisco, CA USA

**Keywords:** Exome sequencing, Copy-number variation, High-performance computing

## Abstract

**Background:**

XHMM is a widely used tool for copy-number variant (CNV) discovery from whole exome sequencing data but can require hours to days to run for large cohorts. A more scalable implementation would reduce the need for specialized computational resources and enable increased exploration of the configuration parameter space to obtain the best possible results.

**Results:**

DECA is a horizontally scalable implementation of the XHMM algorithm using the ADAM framework and Apache Spark that incorporates novel algorithmic optimizations to eliminate unneeded computation. DECA parallelizes XHMM on both multi-core shared memory computers and large shared-nothing Spark clusters. We performed CNV discovery from the read-depth matrix in 2535 exomes in 9.3 min on a 16-core workstation (35.3× speedup vs. XHMM), 12.7 min using 10 executor cores on a Spark cluster (18.8× speedup vs. XHMM), and 9.8 min using 32 executor cores on Amazon AWS’ Elastic MapReduce. We performed CNV discovery from the original BAM files in 292 min using 640 executor cores on a Spark cluster.

**Conclusions:**

We describe DECA’s performance, our algorithmic and implementation enhancements to XHMM to obtain that performance, and our lessons learned porting a complex genome analysis application to ADAM and Spark. ADAM and Apache Spark are a performant and productive platform for implementing large-scale genome analyses, but efficiently utilizing large clusters can require algorithmic optimizations and careful attention to Spark’s configuration parameters.

## Background

XHMM [1] is a widely used tool for copy-number variant (CNV) discovery from whole exome sequencing (WES) data, but can require hours to days of computation to complete for larger cohorts. For example, XHMM analysis of 59,898 samples in the ExAC cohort required “800 GB of RAM and ~1 month of computation time” for the principal component analysis (PCA) component of the algorithm [2]. Substantial execution time and memory footprints require users to obtain correspondingly substantial computational resources and limit opportunities to explore the configuration parameter space to obtain the best possible results.

Numerous algorithms have been developed for WES CNV discovery (see [3] for a review), including the recent CLAMMS [4] algorithm, which was specifically designed for large cohorts. Although XHMM was not specifically designed for large cohorts, the example above shows it is being actively used on some of the largest cohorts in existence. Our focus was to: 1) improve the performance of this widely used tool for its many users; and 2) report on the process of implementing a complex genome analysis for on-premises and cloud-based distributed computing environments using the ADAM framework and Apache Spark.

ADAM is an in-memory distributed computing framework for genome analysis built with Apache Spark [5, 6]. In addition to ADAM, multiple tools, including GATK 4, have (re)implemented genomic variant analyses with Spark [7–14] (see [15] for a review of genomics tools implemented with Spark). The common motivation for using Spark is automatic and generalizable scalability; operations over Spark’s partitioned collections of elements, termed resilient distributed datasets (RDD), can be automatically distributed by the Spark runtime across the available computing resources on a variety of computer systems from multicore workstations to (cloud-based) share-nothing clusters [16]. In contrast, many current genome analysis tools are parallelized by partitioning input files (either physically or via coordinate-sorted indices) stored on a shared file system. Relying on a shared file system for parallel execution introduces I/O overhead, excludes the use of scalable shared-nothing cluster architectures, and makes it difficult to port applications to cloud computing platforms.

**Fig. 1:**
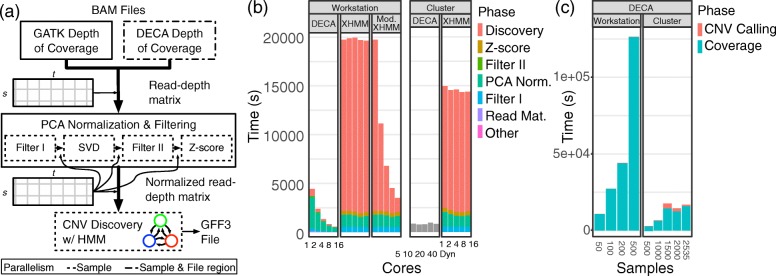
DECA parallelization and performance. **a** DECA parallelization (shown by dashed outline) and data flow. The normalization and discovery steps are parallelized by sample (rows of the samples (s) × targets(t) read-depth matrix). The inputs and outputs of the different components are shown with thinner arrows. **b** DECA and XHMM execution time starting from the read-depth matrix for *s* = 2535 on both the workstation and on-premises Hadoop cluster for different numbers of executor cores. Mod. XHMM is a customized XHMM implementation that partitions the discovery input files and invokes XHMM in parallel. **c** DECA execution time for coverage and CNV discovery for different numbers of samples using the entire workstation (16 cores) and cluster (approximately 640 executor cores dynamically allocated by Spark)

Here we present DECA, a horizontally scalable implementation of XHMM using ADAM and Apache Spark. XHMM is not parallelized, although the user could partition the input files for specific steps themselves and invoke multiple instances of the XHMM executable. In contrast, as shown in Fig. 1a, DECA parallelizes each step of the XHMM algorithm by sample and/or file region to improve execution time compared to the original XHMM implementation and a manually parallelized version of XHMM on a wide variety of computer systems, including in the cloud, while keeping the memory footprint within the resources of a typical compute node (16-256GB). Our secondary goal was to explore the utility of implementing complex genome analyses with ADAM and Apache Spark and report our “lessons learned” parallelizing XHMM with these technologies.

## Implementation

DECA implements the three steps of the XHMM algorithm shown in Fig. 1a: 1) target coverage calculation (to produce the read-depth matrix), 2) PCA normalization and filtering, and 3) CNV discovery by hidden Markov model (HMM) Viterbi decoding. XHMM is designed to use the GATK per-target coverage already calculated as part of a typical genome analysis workflow. DECA can also use a GATK per-target coverage file or can calculate the coverage directly from the original coordinate-sorted BAM files (read via Hadoop-BAM [17]).

DECA implements the XHMM algorithm as a sequence of map, reduce and broadcast operations over RDDs, e.g. the rows of the read depth matrix (each row is a sample) or chunks of a BAM file, which define the operations that are independent and potentially parallelizable. Spark splits this program over RDDs into jobs (all of the actions performed between reading and writing data), splits jobs into stages (all of the actions bound by IO or communication) that must be sequentially executed, and stages into tasks (atomic units of computation which are distributed across the cluster for execution). Spark automatically and transparently partitions those RDDs and the associated computational tasks (expressed as a task graph) across the available computing resources on the different platforms. There is a single DECA implementation used with all platforms, although, as described below, the user may need to adjust the partition sizes (via command line parameters) to achieve the best possible performance on different platforms.

For example, the rows of read-depth matrix (*s* sample*s* × *t* targets) are typically partitioned across the worker nodes and remain resident on a single worker node throughout the entire computation (i.e. computation is sent to the data). Computations over the read depths are performed in parallel on the worker nodes with only summary statistics, e.g. per-target means, communicated between nodes (by reducing from workers to the driver and then broadcasting from the driver to the workers). The first stage of the read depth calculation job reads chunks of the BAM file (via Hadoop-BAM), assigns reads to targets, and local to each task, computes the number of reads assigned to that target from that task. Between the first and second stage, the Spark workers “shuffle” the intermediate counts over the network to co-locate all coverage counts for a given target on the same node before computing the final counts in the second stage (which are either written to storage or consumed by subsequent jobs).

Identifying and removing systematic biases is a key step in WES CNV calling. To do so, XHMM performs singular value decomposition (SVD) on the filtered and centered read-depth matrix (*s* sample*s* × *t* targets) and removes (by default) *K* components with relative variance greater than 0.7 / *n* (for *n* components) that are correlated with systematic biases. Specifically, XHMM removes the *K* components with variance, ${v}_i={\sigma}_i^2$ that satisfy this condition:

$$ {v}_i\ge \frac{0.7\sum v}{n} $$

In practice *K* < < *n*. XHMM computes all *n* components; however, we can identically determine *K* by estimating the total variance from *k < n* components, reducing the time and memory required for SVD. DECA employs a novel iterative algorithm that initially performs SVD with a small *k* (*n /* 10 by default) and increases *k* until the estimate of the total variance is sufficiently precise to determine *K*. For *n* = 2498, for example, DECA computes *k* = 250 components (instead of 2498) to remove *K* = 27. This iterative approach does not change the number of components removed during PCA normalization, or the effect of the normalization step compared to XHMM; instead this algorithmic optimization reduces the computational requirements for determining the number of components to remove.

Specifically, we can estimate the total variance as:

$$ \left(\sum \limits_{i=1}^k{v}_i\right)+\left(n-k-1\right){v}_k $$

**Fig. 2:**
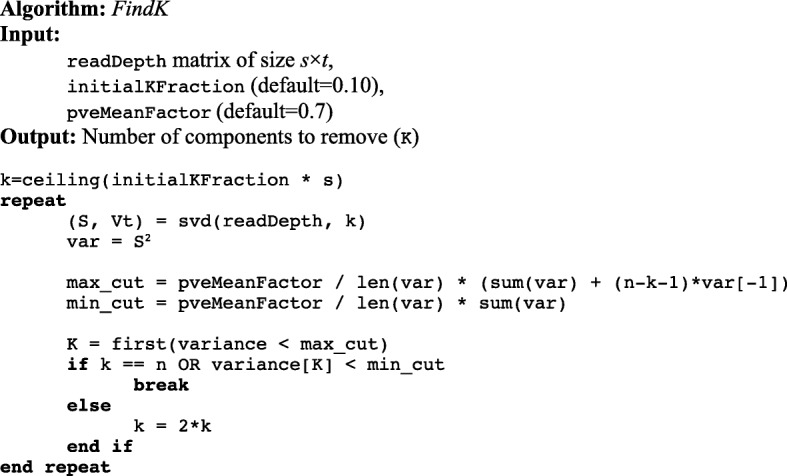
Algorithm for determining *K* components to removing during PCA normalization

Since *v*_*i*_ is monotonically decreasing, our estimate is necessarily greater than but approaching the total variance and thus our estimate for the cutoff to remove components is necessarily greater than but approaching the actual cutoff. Any component with *v*_*i*_ greater than this estimated cutoff will be removed. However, some components with *v*_*i*_ less than the “over” estimate could still also be removed. We can similarly compute a cutoff is that necessarily less than the actual cutoff, i.e. an “under” estimate, by assuming *v*_*i* > *k*_ are 0. If the first component to be retained, i.e. the *K* + 1 component, has variance less than this “under” estimate, then we are guaranteed to have accurately determined K. The algorithm for determining *K* is shown in Fig. 2.

**Fig. 3:**
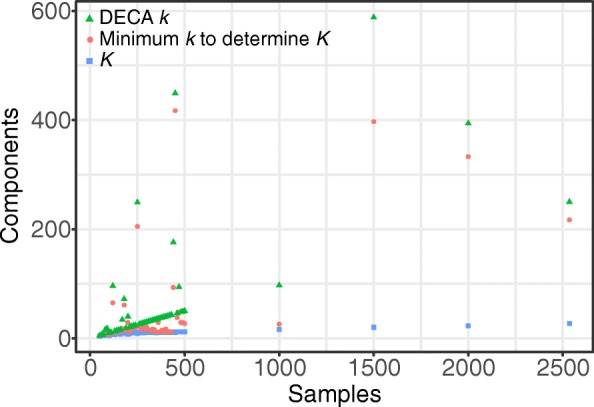
Components to be removed in PCA normalization. *K* components to be removed during PCA normalization, minimum *k* components when computing the SVD to accurately determine *K*, and final *k* used by DECA for different numbers of initial samples for the XHMM default relative variance cutoff of 0.7 / *n*

Figure 3 shows *K*, the number of components to be removed, the minimum *k* to accurately determine *K*, and the actual *k* DECA uses for different numbers of initial samples in the cohort. Although *k* is generally small relative to *n* (less than 10%), for some datasets the minimum *k* to determine *K* can be much larger. Since re-computing the SVD is time consuming, users may consider increasing the initial *k* from the default of 10% of *n* to reduce the chance of needing to compute more components. Tuning the initial *k* is area of ongoing work.

To minimize the required memory for the Spark driver and executors, on a cluster DECA does not collect the entire read-depth matrix, *O(st),* to a single node and SVD is implemented using the distributed algorithm in Spark’s MLlib [18] that requires *O(t)* storage on the executors and *O(kt),* where *k is* typically 0.1 *s,* storage on the driver (at the cost of *O(k)* passes).

To mitigate underflow when multiplying small probabilities in the HMM model, XHMM implements the HMM computation in log-space using the “log-sum-exp trick” and the long double floating point type. DECA similarly implements the Viterbi algorithm in log space, but implements the scaled versions of the forward and backward algorithms [19]. The long double type is not available in the Java Virtual Machine and so all computations in DECA use double precision floating point.

## Results

### Performance evaluation

DECA was evaluated on the on-premises single node and cluster environments described in Table 1 and using Databricks and Elastic Map Reduce on Amazon AWS. Total wall-clock execution time is measured with the time utility. The execution times for individual phases are measured with timing functionality available in the ADAM library. However, due to the lazy construction and evaluation of the Spark task graph, the timing of individual phases is approximate. Specific parameters used for benchmarking are recorded in the source repository. Unless otherwise noted, all benchmarking was performed with DECA commit 0e4a424 and an unmodified copy of XHMM downloaded from the XHMM webpage [20].

**Table 1:** On-premises evaluation systems

Workstation	16-core workstation with two 8-core 2.1 GHz Intel Xeon E5–2620 CPUs, 256 GB RAM, and 16 TB of HDD in 2 × −striped JBOD (four 4 TB 7200 RPM HDDs connected via 6Gbps SATA).
Cluster	56-node Hadoop cluster with 16-core nodes managed by YARN. Each node has two 8-core 2.6 GHz Intel Xeon E5–2670 CPUs, 256 GB RAM and 4 TB of HDD (four 1 TB 7200RPM HDDs connected via 6Gpbs SATA). Nodes are connected with two 1GbE connections and one switchable 10GbE/40Gbps IB connection to a 40GbE TOR switch. HDFS was configured with 128 MB blocks and a 2× replication factor.

We called CNVs in the 1000 Genomes Project phase 3 WES data with *s* = 2535 samples and *t* = 191,396 exome targets [21]. The *s* = 2535 read-depth matrix was generated from the 1000 Genomes Projects phase 3 WES data using GATK DepthOfCoverage [22] according to the XHMM protocol [23] using the target file provided by the 1000 Genomes project. Smaller numbers of samples were obtained by taking subsets of the *s* = 2535 read depth matrix. We excluded targets with extreme GC fraction or low complexity as described in the XHMM protocol. Following the typical usage for XHMM, the read-depth matrix included coverage for all targets and excluded targets were removed during normalization. When performing CNV discovery directly from BAM files with DECA, excluded targets were removed prior to generating the read-depth matrix. All values for user-settable parameters of XHMM were taken from the XHMM protocol.

Figure 1b shows execution time for DECA and XHMM starting from the tab-delimited read-depth matrix. We performed CNV calling on the entire 1000 Genomes phase 3 cohort (*s* = 2535) in 9.3 min on the 16-core workstation (35.3× speedup vs. XHMM) and 12.7 min using 10 executor cores (and 5 driver cores) on the cluster (18.8× speedup vs. XHMM). Note that CNV discovery alone only utilizes a small fraction of the 56-node cluster. DECA could readily scale to much larger cohorts on such a system.

As shown in the execution time breakdown, the speedup is driven by the more efficient HMM model and parallelization of SVD and the HMM model. Using a single workstation core, DECA is approximately 4.4× faster than XHMM. The DECA HMM implementation in isolation is approximately 25× faster than the XHMM HMM on a single workstation core and 325× when using 16 workstation cores.

As noted above, although XHMM itself is not parallelized, the inputs to the CNV discovery phase can be partitioned by the user and the XHMM executable invoked independently on each sub-file. To explore the scaling of this file-based approach, we implemented a parallel wrapper script for XHMM on the workstation. The execution time breakdown is shown in Fig. 1b as “Mod. XHMM”. The modified XHMM is 5.6× faster than single-core XHMM when using 16 workstation cores, while DECA is 7.9× faster than single-core DECA. Overall DECA is 6.3× faster than the modified XHMM when using 16 workstation cores.

Figure 1c shows the total execution time to discover CNVs directly from the coordinate-sorted BAM files for different numbers of samples. DECA can call CNVs from the BAM files for the entire cohort in 4:52 (4 h and 52 min) utilizing up to 640 cores on the cluster. Execution time is dominated by the coverage calculations.

Figure 1c also shows the effect of DECA’s iterative algorithm for PCA normalization (discovery for *s* = 1500 requires more time than *s* = 2000 or *s* = 2535 due to iteratively computing more SVD components) and the performance variability of the shared cluster environment.

DECA can be run unmodified on cloud-based clusters such as Databricks [24] and Amazon AWS’ Elastic MapReduce (EMR), reading from and writing data to Amazon S3. We called CNVs in the full *s* = 2535 cohort starting from the read-depth matrix in 12.3 min using 32 executor cores on Databricks on Amazon AWS with an estimated compute cost of less than $0.35. The Databricks cluster was comprised of four 8-core i3.2xlarge executor nodes and one 4-core i3.2xlarge driver node. We similarly called CNVs on Amazon EMR in 9.8 min using a cluster of four 8-core i3.2xlarge nodes (along with a m4.large master node) with an estimated compute cost of less than $0.35 (not including cluster startup time). We called CNVs directly from the coordinate-sorted BAM files, obtained via the 1000 Genomes public S3 bucket, using 512 executor cores on Amazon EMR in 12.6 h with a compute cost of approximately $225. The EMR cluster was comprised of 64 8-core i3.2xlarge executor nodes and one 4-core i3.2xlarge driver node. We sought to minimize costs for this much larger compute tasks and so used a conservative auto-scaling policy that slowly ramped up the cluster size from 3 to 64 instances over the span of two hours. For all AWS-based clusters we exclusively used spot instances to minimize costs.

### Comparison of DECA and XHMM results

**Fig. 4:**
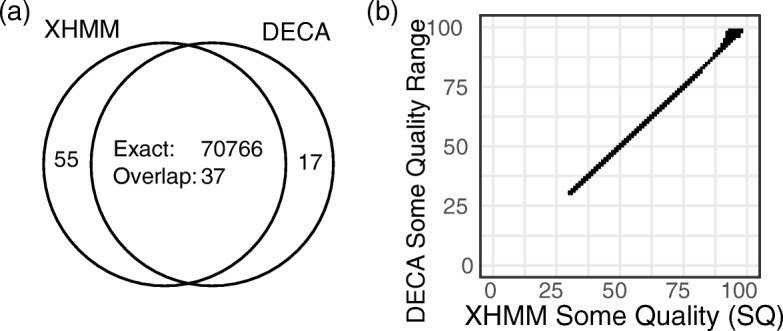
Comparison between DECA and XHMM results. **a** Concordance of XHMM and DECA CNV calls for the full 1000 Genomes Project phase 3 WES dataset (*s* = 2535) when starting from the same read-depth matrix (*t* = 191,396). Exact matches have identical breakpoints and copy number, while overlap matches do not have identical breakpoints. **b** Range of Some Quality (SQ) scores computed by DECA compared to XHMM probability for exact matching variants

Figure 4a shows the comparison of XHMM and DECA CNV calls for the full 1000 Genomes Project phase 3 WES dataset (*s* = 2535) when starting from the same read-depth matrix (*t* = 191,396). Of the 70,858 XHMM calls, 99.87% are called by DECA with identical copy number and breakpoints and a further 37 have an overlapping DECA call with the same copy number. Only 55 XHMM calls do not have an overlapping DECA call. We do not expect identical results between XHMM and DECA due to differences in numerical behavior when multiplying small probabilities in the HMM algorithms.

The 55 XHMM-only events fall into two categories: 1) 50 events spanning just targets 1–3, 2) 5 events with Q_SOME quality scores (the phred-scaled probability that at least one target is deleted or duplicated) at XHMM’s default minimum calling threshold of 30. Most overlapping CNV calls only differ by 1 target (67.6%).

Figure 4b shows a comparison of the XHMM and DECA-calculated quality scores for the 70,766 exactly matching calls. The root mean square (RMS) error in Q_SOME for calls with a XHMM Q_SOME of less than 40 (i.e. those calls close to the calling threshold of 30) is 0.12; the RMS error is 2.04 for all of the calls.

DECA’s coverage calculation is designed to match the GATK DepthOfCoverage command specified in the XHMM protocol. As part of the protocol, the XHMM authors distribute a subset of the 1000 Genomes exome sequencing datasets, specifically reads covering 300 targets in 30 samples. For those 9000 targets, the DECA read-depth differed from the target coverage calculated with GATK 3.7–0-gcfedb67 for only three targets and by less than 0.02.

## Discussion

The primary goal was to make improvements to the performance and scalability of XHMM. Our secondary goal was to explore the utility of building complex genome analyses with ADAM and Apache Spark. Here we report our “lessons learned” re-implementing XHMM with these technologies:

### Library choice matters

XHMM uses LAPACK to perform SVD. The OpenBLAS implementation used here can be several-fold faster than the Netlib reference implementation linked from the XHMM webpage. Table 2 shows the speedup when linking XHMM against OpenBLAS. Switching LAPACK libraries could immediately benefit XHMM users.

**Table 2:** Execution time for XHMM PCA step (--PCA) for different LAPACK libraries. Execution time and speedup for XHMM linked to NetLib and OpenBLAS libraries on the single node workstation using a single core

Samples	NetLib Time (s)	OpenBLAS Time (s)	Speedup
50	9.8	9.5	1.03
500	208.7	112.4	1.86
1000	568.5	241.5	2.35
1500	1150.6	398.5	2.89
2000	2000	585.6	3.42
2535	3178.2	819	3.88

### Spark makes exploiting “embarrassingly parallel” easy and generalizable, but algorithmic optimizations remain important

The initial DECA implementation obtained many-fold speedups, particularly for the “embarrassingly parallel” HMM model where each sample can be analyzed independently. Using Spark MLlib and other libraries we could quickly develop implementations for the PCA normalization and filtering steps that could scale to even larger cohorts. However, without optimizations to reduce *k*, the slower reduced-memory implementation of SVD would reduce possible speedups. Transitioning to a normalized implementation for the HMM forward and backward algorithms and double precision floating resulted in many-fold speedup of the discovery step with minimal differences in the quality scores calculated with those algorithms. The algorithmic optimizations are independent of Spark and could be applied to any XHMM implementation.

### Performance optimization depends on Spark-specific expertise

Improving application performance requires careful attention to distributed programming best practices, e.g. locality, but also Spark-specific expertise such as: RDD caching to avoid re-computation, RDDs vs. Spark SQL (the latter is reported to improve reduce performance, but did not for DECA), and defining performant values for the many Java Virtual Machine (JVM) and Spark configuration parameters to ensure sufficient numbers of tasks, efficient construction of the task graph, and efficient cluster resource utilization.

The two key parameters the user modifies to control concurrency are the number of partitions of the input data and the Spark minimum chunk size for the input. The former determines the minimum number of partitions created when reading the XHMM read-depth matrix from a file and is generally used to increase the number of tasks beyond the number of HDFS blocks (the default partitioning for HDFS files) for computationally intensive tasks. In contrast, when computing the coverage directly from BAM files, the total number of tasks can be in the thousands and needs to be reduced to efficiently construct the task graph. Setting the minimum chunks size larger than the HDFS block size reduces the number of tasks.

## Conclusion

Here we describe DECA, a horizontally scalable implementation of the widely used XHMM algorithm for CNV discovery, which parallelizes XHMM on multicore workstations and large on-premise and cloud-based share-nothing Hadoop clusters using ADAM and Apache Spark. Through a combination of parallelism, novel algorithmic enhancements and other optimizations, DECA achieves a 35-fold speedup compared to the existing XHMM implementation for calling CNVs in the 2535 sample 1000 Genomes exome cohort and can scale to even larger cohorts. By parallelizing all phases of the algorithm, DECA achieves better scaling than approaches based on file partitioning. DECA can be directly deployed on public clouds reducing the need for specialized computational resources to call CNVs in large WES cohorts. We found ADAM and Apache Spark to be a performant and productive platform for implementing large-scale genome analyses, but efficiently exploiting large clusters can require algorithmic optimizations and careful attention to Spark’s many configuration parameters.

## Availability and requirements

Project name: DECA

Project home page: https://github.com/bigdatagenomics/deca

Operating system(s): Platform independent

Programming language: Scala and Java

Other requirements: Maven, Spark 2.1.0+, Hadoop 2.7, Scala 2.11

License: Apache 2

Any restrictions for use by non-academics: None

## Data Availability

The datasets analyzed during the current study are available from the International Genome Sample Resource, http://www.internationalgenome.org.

## Abbreviations

CNV: Copy number variation
HMM: Hidden Markov Model
JVM: Java Virtual Machine
PCA: Principal Components Analysis
RDD: Resilient Distributed Dataset
RMS: Root mean square
SVD: Singular-value Decomposition
WES: Whole exome sequencing
